# Psychosocial and behavioral risk patterns and risk of cardiovascular complications in people with type 2 diabetes

**DOI:** 10.1016/j.diabres.2025.112037

**Published:** 2025-02-09

**Authors:** Xiu Wu, Yuanhao Zu, Danting Li, Yilin Yoshida

**Affiliations:** a Tulane University, School of Medicine, New Orleans, LA 70112, USA

**Keywords:** Psychosocial and behavioral risk patterns, T2D, Cardiovascular complications, Latent class analysis

## Abstract

**Introduction:**

Psychosocial and behavioral risk factors often co-occur in patients with type 2 diabetes (T2D). The clustering of these risk factors and their role in predisposing patients to cardiovascular complications is not well understood. This study aims to identify patient subgroups with distinct psychological and behavioral risk patterns and evaluate the long-term risk of cardiovascular complications associated with these risk patterns.

**Methods:**

A total of 24,467 patients with T2D were identified from the UK Biobank (mean age 59 years, 86.7 % white), used Latent Class Analysis (LCA) to distinguish risk patterns among observed psychosocial (social isolation, loneliness, high neuroticism, anxiety, and depression) and behavioral (smoking, alcohol consumption, sleep duration, diet quality, and physical inactivity) risk factors. the Cox proportional hazards model was applied to assess the association of the identified risk patterns and risk of coronary heart disease (CHD), stroke, and a composite CVD (CHD or stroke) accounting for age, age at T2D diagnosis, race, gender, Townsend Deprivation Index, anti-diabetes medications, lipid-lowering medications, and anti-hypertensive medications.

**Results:**

Three distinct latent classes were identified: a low-risk group (n = 8,227, 33.62 %), a high psychosocial risk group (n = 15,965, 65.25 %), and a high behavioral risk group (n = 275, 1.12 %). Over a median follow-up of 12 years, the fully adjusted model showed that the high psychosocial risk group had a significantly increased risk of CHD (HR = 1.16; 95 % CI 1.08, 1.24) and composite CVD (HR = 1.13; 95 % CI 1.06, 1.20).

**Conclusion:**

The psychosocial risk pattern is significantly associated with the risk of CHD and CVD among patients with T2D. These findings emphasize the importance of integrating psychosocial support into tailored care strategies to mitigate cardiovascular risks in T2D patients.

## 1. Introduction

Type 2 diabetes (T2D) is an established risk factor for cardiovascular disease (CVD). People with T2D have significantly higher cardiovascular morbidity and mortality compared to non-diabetic individuals [1]. Disparities in diabetic cardiovascular complications can be attributed to a variety of risk factors. However, there is a lack of systematic characterization of risk factor patterns, which impedes the understanding of the heterogeneity within T2D to inform effective CVD prevention [2]. In addition to the well-recognized behavioral risk factors, such as smoking, physical activity, diet quality, and weight management, emerging evidence has highlighted the instrumental role of psychosocial risk factors in T2D management and outcomes [3,4]. Psychosocial comorbidities, such as depression and anxiety, are more prevalent in people with T2D than healthy individuals [5]. These comorbidities influence cardiovascular complications through direct physiological effects (e.g., increased cortisol levels and blood pressure) [6] and influences on health behaviors (e.g.,., poor medication adherence and unhealthy lifestyles to cope with stress), which translate to an increased risk of CVD [7,8]. Additionally, chronic social isolation and loneliness, reflecting the quantity and qualityof social relationships, respectively, can trigger prolonged activation of the body’s stress response. This, in turn, leads to sustained elevated cortisol levels and inflammation, ultimately promoting insulin resistance and impairing vascular health [9,10].

Current research on psychosocial and behavioral factors in T2D has often been compartmentalized, with a focus on individual aspects such as glycemic control [11], mental health [12,13], quality of life [14], social and environmental influences [15], and lifestyle factors [16,17]. This fragmented approach has overlooked the complex, interconnected nature of psychosocial and behavioral factors and their collective influence on diabetes outcomes. Furthermore, many of previous studies have been cross-sectional, concentrating on immediate outcomes such as glycemic control that have shed limited insights into how psychosocial and behavioral factors contribute to long-term diabetic complications. To address these gaps, this research utilized data from the UK Biobank (UKB), which includes a diverse cohort of diabetes patients who underwent a broad spectrum of psychosocial and behavioral risk assessments at baseline and with adjudicated CVD outcomes over ten years of follow-up. The study aimed to identify underlying clusters of T2D patients with distinct psychological and behavioral risk patterns and to assess the long-term risk of cardiovascular complications associated with these patterns.

## 2. Methods

UKB is a prospective cohort study that enrolled approximately half a million participants. Individuals aged 40–69 years were recruited between 2006 and 2010 [18]. Our analysis included a total of 24,467 individuals with T2D and without CVD history who have completed behavioral and psychosocial assessments via touchscreen surveys. Details on participant selection are provided in the supplemental materials. T2D was defined based on a self-reported physician’s diagnosis, current use of insulin or oral hypoglycemic medications, or an HbA1c level of ≥6.5 % [19]. (see Data Selection in Supplemental Fig. 1).

### 2.1. Psychosocial risk factors

Social isolation, loneliness, neuroticism, depression, and anxiety were assessed using validated questionnaires in UKB [20–22] (See detailed categorizations in Supplemental Table 2). Social isolation is assessed using two questions: “How often do you visit friends or family or have them visit you?” and “Which of the following [leisure/social activities] do you engage in once a week or more often?” We categorized those who visited friends and family less than once a month as socially isolated [23]. Loneliness is assessed using two questions: “Do you often feel lonely?” and “How often are you able to confide in someone close to you?”. We categorized those who reported “lonely” and “once every few months to never or almost never to confide” into the lonely group [24]. Neuroticism score, reflecting an individual’s emotional instability, anxiety, moodiness, and negative feelings, was measured by the 12-item Eysenck Personality Questionnaire-Revised Short Form [22]. We categorized those with >=3 as high neuroticism [25]. Depression and anxiety were measured by the Patient Health Questionnaire (PHQ-4) [22]. Depression was assessed through two questions: “Frequency of depressed mood in the past two weeks” and “Frequency of unenthusiasm or disinterest in the past two weeks.” Anxiety was evaluated with two questions: “Frequency of tenseness or restlessness in the past two weeks” and “Frequency of tiredness or lethargy in the past two weeks.” We categorized those reported having symptoms “More than half the days” or “nearly every day” as depression or anxiety [26].

### 2.2. Behavioral risk factors

Behavioral risk factors included smoking, alcohol consumption, physical activity, sleep duration, overall diet quality, and obesity [17,27]. Smoking status was categorized as never, former (who have quit but had consumed more than 100 cigarettes in a lifetime), or current smoker [28]. Physical activity was evaluated based on the Physical Activity Guidelines for Americans, with 8.3 MET (Metabolic Equivalent of Task) hours per week serving as the threshold [29]. The diet quality score was defined based on total fruit intake (>=4.5 pieces per week), total fish intake >=2 servings per week, processed meat intake < twice per week, and red meat intake <=5 times per week [30], and total vegetable intake used cutoff values 8 tablespoons [31]. It was calculated by summing the individual scores of the five factors, with a range of 0–5 [31]. A score < 4 indicates poor diet quality. Obesity, an indicator of weight management, was defined by BMI > 30 [3]. (See detailed variable definitions and coding schemes in Supplemental Description and Supplemental Table 2).

### 2.3. Outcomes

Baseline participants were from hospital inpatient records to gather information on admissions, diagnoses, and deaths. The main outcomes of this study were the CVD events, including CHD and stroke. Outcomes were identified based on ICD-10 codes, with CHD classified under codes I20–I25 and stroke under codes I60–I64. Follow-up time was measured from the baseline date until the diagnosis of the outcome, death, or the censoring date (May 23, 2021), whichever came first. Additional details on outcome ascertainment can be found online at https://biobank.ctsu.ox.ac.uk/showcase/label.cgi?id = 2000.

### 2.4. Statistical analysis

1. Data processing
  In this study, 11 exploratory variables for Latent Class Analysis (LCA, see below) and other covariates were selected based on prior research highlighting their significant adverse effects on T2D management and outcomes [17,27,32,33]. To address missing data, we implemented a multivariate imputation strategy for variables with over 10 % missing data, including physical activity, diet, depression, anxiety, neuroticism, loneliness, dyslipidemia, and hypertension. The imputation was done by a multivariate C50 decision tree model, which effectively preserves the original dataset distribution [34].
2. LCA
  LCA, a clustering approach, was employed to distinguish distinct groups of patients exhibiting similar behaviors and psychosocial factors [35]. Models ranging from one to six classes were evaluated using LCA in Mplus to determine the optimal model fit for identifying risk profiles in T2D patients. The selection criteria included minimization of Bayesian Information Criterion (BIC) and Akaike Information Criterion (AIC) values, higher entropy values (indicating better classification accuracy), and posterior probabilities (close to 1 reflecting high probability of correct classification for each patient into a specific class). *(Selection criteria and results are presented in* Supplemental Table 1*)*.
3. Schoenfeld residuals test
4. Cox proportional hazard models
  Cox proportional hazard models were employed to assess the association between the identified risk patterns and CVD outcomes. The proportional hazards assumption was evaluated using Schoenfeld residuals, and the results confirmed that all analyses satisfied the required assumptions. In the multivariable-adjusted model, Models were adjusted for age, sex, race, deprivation index, and age at diabetes diagnosis and further adjusted for medication factors (e.g., hypertension medications, antidiabetic medications, and lipid-lowering medications). The analysis was performed by R (RStudio/2024.04.2).

## 3. Results

### 3.1. Data description

Table 1 presents the characteristics of UKB participants with T2D. 87 % of participants were white, 8694 were women, and mean age at recruitment was 59 years. Mean diagnosed age was 49 years. Additionally, participants with T2D had high rates of use of medication for chronic conditions. The Townsend Deprivation Index was negative, indicating relative affluence. In terms of psychosocial factors, 10.4 % of participants experienced social isolation, 9 % reported loneliness, 12.5 % had depression, 21.5 % suffered from anxiety, and 66.1 % exhibited high levels of neuroticism. The prevalence of behavioral risk factors among participants with T2D included current smoking (11.3 %), frequent alcohol consumption (53.6 %), physical inactivity (42.1 %), short sleep duration (28.1 %), moderate to poor diet quality (66.7 %), and obesity (53.6 %).

### 3.2. LCA results

**Fig. 1.**
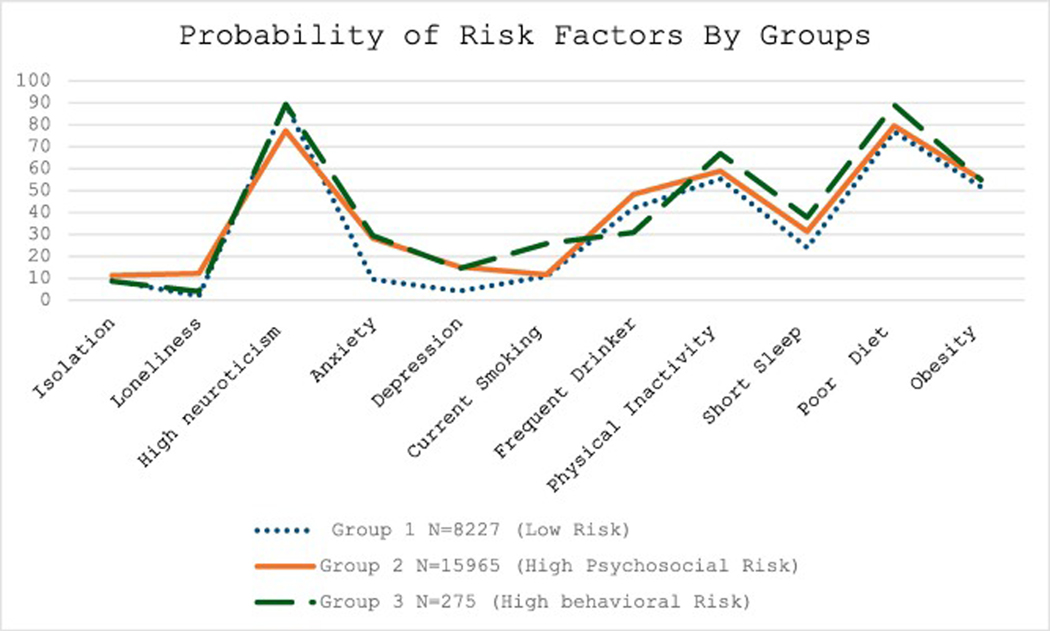
The Probabilities of Risk Factors by Groups.

Fig. 1 and Table 2 illustrate the probabilities of risk factors by identified risk pattern (or group). Group 1,’low risk group’, includes 8,227 patients who exhibited lower probabilities of both psychosocial and behavioral risk factors relative to the other two groups. Group 2, the ‘high psychosocial risk group,’ consisted of 15,965 patients exhibited high probabilities of nearly all psychosocial risk factors. Group 3, ‘high behavioral risk group,’ included 275 patients and exhibited the highest behavioral risks. This group also has a younger diagnosed age (<45 years) and higher deprivation score (Table 2).

### 3.3. Proportional hazards Regression results

Table 3 shows the Cox regressions for the associations between the latent groups and incident cardiovascular complications. In the unadjusted model, the hazard ratios for CHD, stroke, and composite CVD were not statistically significant. However, after adjusting for demographic factors, the high psychosocial risk group demonstrated significantly increased risks for CHD (HR = 1.15; 95 % CI: 1.07–1.23) and overall CVD (HR = 1.12; 95 % CI: 1.06–1.20). In the fully adjusted model, the risks for CHD (HR = 1.16; 95 % CI: 1.08–1.24) at high psychosocial risk group, the risks for CHD (HR = 1.21; 95 % CI: 1.07–1.23) at the high behavioral risk group and CVD (HR = 1.13; 95 % CI: 1.06–1.20) remained significant.

## 4. Discussion

Based on a large cohort of individuals with T2D that assessed on a wide variety of risk factors, we identified three distinct risk patterns: a high psychological risk group, a high behavioral risk group, and a low-risk group. In the prospective analysis, we found that being in the psychosocial risk group was associated with a significantly elevated risk of CHD and composite CVD outcome relative to being in the low-risk group. The behavioral risk group was associated with a significant risk of CHD but not with the composite CVD compared to those being in the low-risk group.

The adverse impact of psychosocial risk factors, including social adversities and psychological comorbidities, on T2D management and outcomes has been indicated in previous research [36–44]. Social isolation and loneliness disproportionately affect T2D patients compared to healthy individuals. Liang et al. (2023) reported that an estimated 38 % of T2D patients experience social disconnection, significantly higher than the 7–10 % observed in the general population. One key reason for this disparity is that chronic conditions often limit individuals’ ability to engage in social interactions, leading to isolation or disconnection from others [36]. Simultaneously, managing a chronic illness, worrying about long-term health complications, and bearing the burden of self-care can exacerbate persistent psychological stress. This leads to increased levels of cortisol and other stress hormones, which in turn heighten insulin resistance and elevate blood pressure, thereby increasing the risk of CVD [37–40]. Additionally, the absence of inter-personal connections can lead to diminished health-seeking behaviors among individuals with T2D. Isolation often leaves patients without a support system, such as family or friends, who would typically encourage and assist them in seeking medical care [41]. As a result, these patients may face several challenges, including transportation difficulties, navigating healthcare systems, and missing essential medical appointments. These barriers collectively contribute to suboptimal T2D management, further aggravating the risk of CVD in T2D patients [42–44].

T2D individuals have higher psychological comorbidities, such as depression and anxiety, than healthy individuals, which also have a negatively multifaceted impact on cardiovascular complications. In addition to contributing to poor health behaviors such as poor weight management [45] and glycemic control [46], their psychosocial comorbidities elevate the release of stress hormones, leading to increased CVD risk through a number of mechanisms [47]. Depression and anxiety can cause dysregulation of the autonomic nervous system, characterized by reduced heart rate variability and heightened sympathetic nervous system activity [48]. This imbalance can promote the development of atherosclerosis, arrhythmias, and other cardiovascular complications. Additionally, these conditions are often linked to elevated levels of inflammatory markers such as C-reactive protein (CRP) and interleukin-6 (IL-6), which are associated with both CVD and T2D. Chronic inflammation is a key factor in the development of atherosclerosis and related diseases [49].

The identification of a distinct psychosocial pattern and its association with risk of CVD among patients with T2D underscores the critical need for targeted interventions to mitigate CVD risks. Healthcare providers should consider implementing comprehensive psychosocial support, including individualized psychological counseling, stress management workshops, and cognitive-behavioral therapy (CBT) to enhance psychosocial resilience [50]. Additionally, establishing community support networks (e.g., telehealth utilization in diabetes care) can significantly alleviate feelings of isolation and loneliness [51]. Tailored psychosocial support and behavioral modification programs are also essential to reduce cardiovascular complications, as psychosocial and behavioral risk factors often interact and collectively influence health outcomes [52]. By proactively adopting these strategies, patients with T2D can have improved health outcomes and the overall quality of life.

The strengths of this research include leveraging a large, longitudinal cohort, which supports sufficient power for LCA and a prospective association for the identified risk patterns and the risk of CVD. The richly phenotyped also enables us to account for various covariates to minimize confounding effects. However, several limitations must be acknowledged. First, UKB is predominately represented by Caucasian white participants. The generalizability of the findings needs to be substantiated in cohorts with diverse racial/ethnic background. Additionally, since the psychosocial and behavioral variables were only measured at baseline, the results may not reflect changes over time. Third, the results for the association between behavioral risk group membership and risk of CVD were statistically significant, potentially due to the small sample size of this group.

## 5. Conclusion

Our study identified distinct psychological and behavioral risk patterns among patients with T2D. The resultshighlighted the significant association between CVD risk and membership in the high psychosocial risk group. Our study underscores the importance of psychosocial support in enhancing cardiovascular outcomes for individuals with T2D.

## Supplementary Material

Supplementary Material

Appendix A. Supplementary data

Supplementary data to this article can be found online at https://doi.org/10.1016/j.diabres.2025.112037.

## Figures and Tables

**Table 1:** Characteristics of UKB participants with T2D.

*Sociodemographic Factors*	All (24,467)
Age, years (mean, SD)	59 (7.4)
T2D diagnosis age (mean, SD)	49.6 (14.8)
White	21,219 (86.7)
Non-White	3037 (13.3)
Women	8694(86.01)
Townsend deprivation index (mean, SD)	−0.48 (3.4)
Hypertension medications	19107(78.1)
Dyslipidemia medications	19,754 (80.7)
Anti-diabetes medications	20,897 (85.4)
***Psychosocial Risk Factors***	
Social isolated	2550 (10.4)
Loneliness	2138 (9)
Depression (PHQ-4)	2819 (12.5)
Anxiety (PHQ-4)	5254 (21.5)
High neuroticism (score > 70 percentile)	16,173 (66.1)
***Behavioral Risk Factors***	
Current smoker	2743 (11.3)
Frequent drinker (Daily or 2–4 x/week)	13,057 (53.6)
Physical inactivity (< recommended moderate/vigorous MET)	10,297 (42.1)
Short sleep duration (<7 h/night)	6769 (28.1)
Moderate/poor diet quality (diet score < 4)	16,332 (66.7)
Obesity	13,004 (53.6)

**Table 2:** Probability of Classification Factors and Sociodemographic Characteristics across Psychosocial and Behavioral Groups.

	group 1 N = 8227 (relatively healthy)	group 2 N = 15965 (high psychosocial risk)	Group 3 N = 275 (high behavioral risk)
**Probability of Classification factors**			
Social isolation	8.9	11.3	8.7
Loneliness	2	12.3	4
High neuroticism	89	77	89
Anxiety	9.5	28.3	29.5
Depression	4.2	15.2	14.5
Current Smoker	11	11.7	25.8
Frequent Drinker	42	48.3	30.9
Physical Inactivity	55.3	58.9	66.9
Short Sleep	24	31.4	37.8
Moderate/poor diet quality	76.8	79.5	89.1
Obesity **Demographic covariates**	51.7	55.1	54.9
Age (mean, SD)	59.9 (7.0)	58.5 (7.5)	56.3 (8.3)
Diagnosed age (mean, SD	50.7 (14.2)	49.1(15)	43.0 (20.4)
Non-white (n, %)	992 (12.1)	2091 (13.1)	165 (60.0)
Women (n, %)	2832 (34.4)	7243 (45.4)	128 (46.6)
Townsend deprivation index (mean)	−1.02 (5.1)	−0.474 (5.2)	1.8 (3.4)
Anti-diabetes medications (n, %)	4955 (60.23)	9558 (59.87)	172 (62.55)
Lipid-lowering medications (n, %)	6595 (80.16)	12,910 (80.86)	249 (90.55)
Anti-hypertensive medications (n, %)	6399 (77.78)	12,471 (78.11)	237 (86.18)

**Table 3:** Cox regressions for the association of psychosocial-behavioral risk patterns and risk of CVD.

	Incidents /population	Unadjusted HR (95 %CI)	Demographic Adjusted HR # (95 %CI)	Fully Adjusted HR## (95 %CI)
**CHD**				
Low-risk (group 1)	1308/8227	Ref.	Ref.	Ref.
High psychosocial risk (group 2)	2674/15965	1.06 (0.99, 1.13).	**1.15 (1.07, 1.23)** ***	**1.16 (1.08, 1.24)** ***
High behavioral risk – young dx (Group 3)	47/275	1.10 (0.82, 1.48)	1.11 (0.75, 1.66)	**1.21 (1.07, 1.23)** ***
**Stroke**				
Low-risk (group 1)	344/8227	Ref.	Ref.	Ref.
High psychosocial risk (Group 2)	628/15965	0.93 (0.82, 1.07)	1.01 (0.88, 1.16)	1.02 (0.89, 1.17)
High behavioral risk – young dx (Group 3)	9/275	0.81 (0.42, 1.57)	1.03 (0.46, 2.30)	1.01 (0.45, 2.27)
**All CVD**				
Low-risk (Group 1)	1725/8227	Ref.	Ref.	Ref.
High psychosocial risk (Group 2)	3463/15965	1.04 (0.98, 1.10)	**1.12(1.06, 1.20)** ***	**1.13 (1.06, 1.20)** ***
High behavioral risk – young dx (Group 3)	58/275	1.03 (0.79, 1.34)	0.95 (0.65, 1.38)	1.03 (0.72, 1.48)

Note:

# Adjusted model accounted for age, sex, race, diagnosed age, Townsend Deprivation Index.

## Additionally adjusted for anti-hypertension medications and lipid-lowering medications, and anti-diabetic medications.

*** P < 0.0001.
