# Supplementary material for: Psychosocial and behavioral risk patterns and risk of cardiovascular complications in people with type 2 diabetes
Source: Diabetes Res Clin Pract. Author manuscript; Available in PMC 2025 Sep 1. (PMC12203623; doi:10.1016/j.diabres.2025.112037)
Supplement: Supplementary Material [file NIHMS2086577-supplement-Supplementary_Material.docx]

### **Supplement**

**Data selection**

Individuals aged 40–69 years were recruited between 2006 and 2010 [1]. After excluding 36,001 participants with missing values on HbA1c, 18,028 participants with a history of stroke and coronary heart disease (CHD), and 3201euglycemic and 420808 pre-diabetes in Figure 1, 24,467 individuals were selected in the main analysis.

All UKB participants N=502,505

Missing HbA1c N=36,001

Prevalent Stroke or CHD N=18,028

pre

CVD-free with complete glycemic measures N=448,476

Euglycemic N=3,201

Pre-DM N=420,808

All CVD-free T2D N=24,467

Figure 1 Flow Chart of Data Selection.

**Schoenfield residuals**


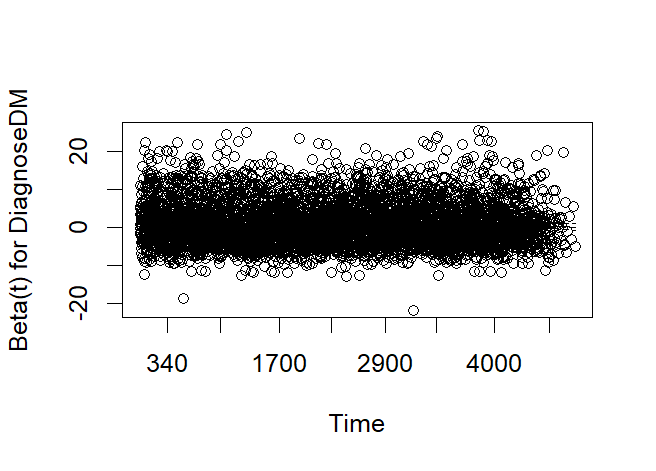


Figure 2 CVD Schoenfield Residuals Test.

**CVD Kaplan-Meier curve**

We have visualized the survival differences for CVD using Kaplan-Meier curves.

**
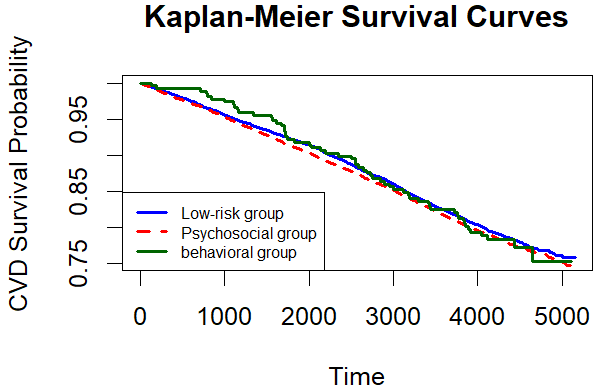
**

Figure 3 CVD Kaplan_Meier curve.

**LCA procedure**

According to Table 1, a group of 3 clusters has the highest entropy of 1 and the lowest likelihood of -12440. The AIC and BIC were lower than others. We, therefore, chose a number of 3 clusters (risk patterns)

Table 1 Model fit indicators for LCA from 1 to 6 classes.

| Models | LL | AIC | BIC | CAIC | Smallest class count (n) | Entropy | VLMR-LRT |
| --- | --- | --- | --- | --- | --- | --- | --- |
| Group 1 | -651169 | 1302397 | 1302632 | 1302660.723 | 0 | - | - |
| Group 2 | -168404 | 336891.5 | 337231.9 | 337273.9294 | 275 | 1 | 0 |
| Group 3 | -124401 | 248931.4 | 249458.2 | 249523.1902 | 275 | 1 | 0.299 |
| Group 4 | -330350 | 660923 | 661828 | 661940.1298 | 1683 | 0.899 | 0 |
| Group 5 | -107320 | 214862.8 | 215762.5 | 215873.5079 | 89 | 1 | 0.2678 |
| Group 6 | -109451 | 219170 | 220256.1 | 220390.0528 | 1 | 1 | 1 |

Note: AIC Akaike’s information criterion, BIC Bayes information criterion, CAIC consistent AIC.

**Codes**

All variable codes were shown in Table 2.

**Psychosocial Risk Factors**

Social isolation is coded as follows: if the value is greater than 4 (indicating visits from friends and family occur less than once a month), it is coded as 1 (isolated). If the value is greater than 0 but less than or equal to 4 (indicating visits occur more than once a month), it is coded as 0 (not isolated). If both questions receive a value of 1, the participant is coded as 1 (isolated). If one or both values are 0, the participant is coded as 0 (not isolated).

Loneliness is assessed using two questions: "Do you often feel lonely?" and "How often are you able to confide in someone close to you?" If the response to either question is 1 (indicating loneliness), loneliness is coded as 1 (lonely). If neither response equals 1, it is coded as 0 (not lonely). Depression, anxiety, and neuroticism are evaluated using consistent coding criteria. values exceeding 3 (indicating symptoms present "more than half the days" or "nearly every day") are coded as 0, signifying the presence of depression, anxiety, or neuroticism. Values of 3 or less are coded as 1, indicating the absence of these conditions.

**Behavioral risk factors**

Behavioral risk factors include smoking, alcohol consumption, physical activity, sleep duration, overall diet, and obesity.

Smoking status is classified into three categories: never, former, and current smoker. Never smokers are coded as 0, former smokers as 1, and current smokers (those who have smoked 100 or more cigarettes) as 2. Alcohol consumption is categorized into two groups based on intake frequency: frequent drinkers, defined as those who drink "daily or almost daily," "three or four times a week," or "once or twice a week" (coded as 1), and less frequent drinkers, defined as those who drink "one to three times a month," "on special occasions only," or "never" (coded as 0).

Physical activity is categorized as "physically active" (coded as 1) for individuals who meet or exceed 8.3 MET hours per week, and "physically inactive" (coded as 0) for those who do not. Sleep duration is treated as a continuous variable, with individuals sleeping fewer than 7 hours per night (but more than 0 hours) coded as 0, while those who sleep 7 or more hours per night are coded as 1. Obesity is defined by a body mass index (BMI) of 30 or higher, with individuals classified as obese (coded as 1) if their BMI is 30 or above, and non-obese (coded as 0) if their BMI is below 30.

A diet score above 4 was coded as 0, indicating an ideal diet, while a score between 0 and 4 was coded as 1, indicating a moderate or poor diet. Vegetable and fruit intake included cooked vegetables, raw vegetables, fresh fruit, and dried fruit. If both categories (vegetables and fruit) had a value of 0, the intake was coded as 0; otherwise, it was coded as 1. Fish intake included both oily and non-oily fish, with the following frequency coding: "less than once a week" was coded as 0.5, "once a week" as 1, "5-6 times a week" as 5, and "daily or more" as 7. If the combined fish intake exceeded a frequency of 2 per week, it was coded as 1 (adequate intake); if the intake was 2 or fewer times per week, it was coded as 0 (inadequate intake). Processed meat intake was coded as 1 if consumed less than 3 times per week, indicating a moderate or poor diet, and coded as 0 if consumed 3 or more times per week, indicating an ideal diet.

Red meat intake, which included poultry, beef, lamb, and pork, followed the same frequency coding as fish intake: "less than once a week" was coded as 0.5, "once a week" as 1, "5-6 times a week" as 5, and "daily or more" as 7. If the total red meat intake was 5 times per week or fewer, it was coded as 1 (moderate/poor diet); if consumed more than 5 times per week, it was coded as 0 (ideal diet).

**Covariates**

Self-reported data were collected using a touchscreen questionnaire, covering variables such as recruitment age, race (categorized as white and non-white), Townsend Deprivation Index, obesity (BMI ≥ 30), baseline hypertension (use of antihypertensive medications), baseline dyslipidemia (use of lipid-lowering medications), baseline antidiabetic medications (classified as insulin use, oral antidiabetic drugs only, or neither), and age at diabetes diagnosis, and anti-diabetes in Table 2. The Townsend Deprivation Index is a composite measure of socioeconomic deprivation, based on factors such as unemployment, lack of car ownership, lack of home ownership, and household overcrowding [2]. Information on diabetes medications was obtained from both self-reported data and nurse-led verbal interviews [3].

Table 2 Covariates and Explanatory Variables List

| Variable | Description | UKB Field_ID | Coding |
| --- | --- | --- | --- |
| Age | Age at recruitment | n_21022_0_0 |  |
| White | Ethnic background | n_21000_0_0 | if n_21000_0_0=1 then white=1; else if n_21000_0_0>. then white=0  1 white, 2 asian, 3 black,4 mixed and other |
| Sex | Sex | n_31_0_0 | Our categories: 0(Female), 1(Male) |
| Sleep duration | the total amount of sleep a participant gets, either during one night or over a 24-hour period. | n_1160_0_0; n_1160_1_0; n_1160_2_0; n_1160_3_0 | Our categories: 0(<7), 1(>=7) |
| Alcohol | Alcohol intake frequency. | n_1558_0_0 | 1 Daily or almost daily  2 Three or four times a week 3 Once or twice a week 4 One to three times a month 5 Special occasions only 6 Never Our categories: frequent drinker (1-3 ), less frequent drinker (4-6) |
| DM diagnosis age | Age diabetes diagnosed | n_2976_0_0 |  |
| Townsend deprivation index | a measure of socioeconomic deprivation at the area level in the UK Biobank | n_22189_0_0 |  |
| smoking | Participants self-reported smoking status | n_20116_1_0~n_20116_3_0 | Our categories: 0(Never), 1(Previous), 2(Current) |
| Physical Activity | Summed MET minutes per week for all activity | n_22035_0_0 | Our categories: 0(No), 1(Yes) |
| Baseline hypertension | hypertension medication | n_6177_0_0,n_6177_0_1,n_6177_0_3, n_6153_0_0, n_6153_0_1, n_6153_0_2 | if n_6177_0_0=2 \| n_6177_0_1=2 \| n_6177_0_2=2 \| n_6153_0_0=2 \| n_6153_0_1=2 \| n_6153_0_2=2 \| n_6153_0_3=2 then Baseline hypertension=1 |
| Baseline dyslipidemia | dyslipidemia medication | n_6177_0_0, n_6177_0_1, n_6177_0_3,  n_6153_0_0,  n_6153_0_1,  n_6153_0_3 | if n_6177_0_0=1,3 \| n_6177_0_1=1,3 \| n_6177_0_2=1,3 \| n_6153_0_0=1,3 \| n_6153_0_1=1,3 \| n_6153_0_2=1,3 \| n_6153_0_3=1,3 then Baseline dyslipidemia=1 |
| Social Isolation | Frequency of visit friends/family and leisure/social activity | n_1031_0_0; n_6160_0_0 | If n_1031_0_0>=4 isolation=1 "friends and family visit less than once a month"  If n_1031_0_0>0 and n_1031_0_0<4 isolation=0 "friends and family visit beyond once a month" ;   If n_6160_0_0 >=1 activity=0 " participate 1more activity"  If n_6160_0_0=-7 activity =1 "no participation in social activities at least weekly “Social Isolation=1 isolated (score=2) If "friends and family visit beyond once a month"=1 and "no participation in social activities at least weekly" =1,otherwise, Social Isolation=0 not isolated (1 or 0) |
| Loneliness | ACE touchscreen question "Do you often feel lonely?" | n_2020_0_0; n_2110_0_0 | If n_2020_0_0=1 "lonely" If n_2020_0_0=0 "not lonely" If n_2110_0_0>=0 and n_2110_0_0<2 "once every few months to never or almost never"=1 If n_2110_0_0>=2 "almost daily to about once a month"=0 If "lonely" & "once every few months to never or almost never"=1 Loneliness=1 If "not lonely" or "almost daily to about once a month" Loneliness=0 |
| Neuroticism score (0-12) | a personality trait that is defined by a tendency to experience negative emotions. | n_20127_0_0 | Our categories: 1(>=3), 0(0,3) |
| Depression | a range of personality and psychiatric disorders. | n_2050_0_0; n_2060_0_0 | Our categories: 1(>=3), 0(0,3) |
| Anxiety | Experience of pain | n_2070_0_0; n_2080_0_0 | Our categories: 1(>=3), 0(0,3) |
| diet | summing the intake of vegetables, fruit, fish, processed meat, and red meat | n_1359_0_0; n_1369_0_0; n_1379_0_0; n_1389_0_0 | 0 Never 1 Less than once a week 2 Once a week 3 2-4 times a week 4 5-6 times a week 5 Once or more daily If total score > 4, diet=0;If a score in (0, 4), diet=1 |
| Obesity | Body mass index (BMI>=30) | n_21001_0_0 | Our categories: 1(>30), 0(0,30) |

**Reference**

1. Sudlow C, Gallacher J, Allen N et al (2015) UK Biobank: an open access resource for identifying the causes of a wide range of complex diseases of middle and old age. PLoS Med 12(3): e1001779. <https://doi.org/10.1371/journal.pmed.1001779>
2. Yousaf S, Bonsall A. UK Townsend Deprivation Scores from 2011 Census Data. Colchester, UK: UK Data Service; 2017.
3. Eastwood SV, Mathur R, Atkinson M, Brophy S, Sudlow C, Flaig R, et al. Algorithms for the capture and adjudication of prevalent and incident diabetes in UK Biobank. PLoS One 2016;11:e0162388. <https://doi.org/10.1371/journal.pone.0162388>
